# Comprehensive analysis of immunogenic cell death-related genes in liver ischemia-reperfusion injury

**DOI:** 10.3389/fimmu.2025.1545185

**Published:** 2025-02-17

**Authors:** Kai Lu, Hanqi Li, Liankang Sun, Xuyuan Dong, Yangwei Fan, Danfeng Dong, Yinying Wu, Yu Shi

**Affiliations:** ^1^ Department of Hepatobiliary Surgery, The First Affiliated Hospital of Xi’an Jiaotong University, Xi’an, China; ^2^ Department of Oncology, The First Affiliated Hospital of Xi’an Jiaotong University, Xi’an, China

**Keywords:** liver ischemia-reperfusion injury, immunogenic cell death, machine learning, hub genes, diagnosis model

## Abstract

**Background:**

Liver ischemia-reperfusion injury (LIRI) is a critical condition after liver transplantation. Understanding the role of immunogenic cell death (ICD) may provide insights into its diagnosis and potential therapeutic targets.

**Methods:**

Differentially expressed genes (DEGs) between LIRI and normal samples were identified, and pathway enrichment analyses were performed, followed by immune infiltration assessment through the CIBERSORT method. The consensus clustering analysis was conducted to separate LIRI clusters and single-sample Gene Set Enrichment Analysis (ssGSEA) was used to analyze the distinct immune states between clusters. Weighted Gene Co-Expression Network Analysis (WGCNA) was employed to identify hub genes associated with ICD. To establish diagnostic models, four machine learning techniques, including Random Forest (RF), XGBoost (XGB), Support Vector Machine (SVM), and Generalized Linear Models (GLM), were applied to filter gene sets. The receiver operating characteristic (ROC) curves were utilized to assess the performance of the models.

**Results:**

Pathway enrichment results revealed significant involvement of cytokines and chemokines among DEGs of LIRI. Immune infiltration analysis indicated higher levels of specific immune functions in Cluster 2 compared to Cluster 1. WGCNA identified significant modules linked to LIRI with strong correlations between module membership and gene significance. The RF and SVM machine learning algorithms were finally chosen to construct the models. Both demonstrated high predictive accuracy for diagnosing LIRI not only in training cohort GSE151648 but also in validation cohorts GSE23649 and GSE15480.

**Conclusions:**

The study highlights the pivotal roles of ICD-related genes in LIRI, providing diagnosis models with potential clinical applications for early detection and intervention strategies against LIRI.

## 1 Introduction

Liver transplantation is a vital therapeutic option for patients suffering from end-stage liver disease and acute liver failure (1). As the prevalence of chronic liver diseases such as hepatitis, cirrhosis, and fatty liver disease continues to rise globally, the demand for liver transplants has reached unprecedented levels (2). The success of liver transplantation is contingent upon numerous factors, including donor organ quality, surgical technique, and postoperative care (3). However, one significant complication that adversely affects outcomes is liver ischemia-reperfusion injury (LIRI) (4).

In the context of liver transplantation, LIRI can lead to a cascade of pathological events that result in hepatocyte injury, inflammation, and apoptosis, ultimately compromising graft function (5). The severity of LIRI is influenced by several factors, including the duration of ischemia, the condition of the donor organ, and the recipient’s immunological status (6). Complications arising from LIRI include acute liver failure, prolonged hospitalization, and chronic rejection or fibrosis, making it a central priority in the continuous investigation of liver transplantation (7).

LIRI is closely associated with the immune system and the inflammatory reaction in the liver mediated by immune cells in response to injury (8). It is well established that immune responses and inflammation are intricately associated with the pathogenesis and outcomes of LIRI. Recently, several studies have demonstrated that the activation of innate and adaptive immune system is an essential event in development of LIRI (9–13). A critical pathophysiological mechanism underlying LIRI is its association with immunogenic cell death (ICD) (14). Immunogenic cell death is defined as a form of cell death that induces a potent immune response, thereby contributing to the generation of adaptive immunity (15). ICD can occur through various modalities, including apoptosis, necroptosis, and pyroptosis, each characterized by distinct biochemical and morphological features (16). The release of damage-associated molecular patterns (DAMPs) during these cell death processes plays a crucial role in activating the immune system and promoting inflammation (17). In LIRI, the interplay between ICD and the immune response can exacerbate tissue injury and influence the subsequent immune tolerance of the transplanted liver.

Understanding the role of ICD in LIRI is of great importance for several reasons. First, elucidating the specific genes and molecular pathways involved in ICD can provide insights into the mechanisms that govern liver injury and repair. Second, targeting these pathways could lead to the development of novel therapeutic strategies aimed at mitigating LIRI, thereby improving graft survival rates and patient outcomes. Recent studies have highlighted the potential strategy for pharmacological interventions that modulate immunogenic cell death, suggesting that a deeper understanding of ICD could help guide future clinical applications (18, 19).

However, there remains a significant gap in understanding the role of ICD in liver ischemia-reperfusion injury. Therefore, our study undertook a thorough analysis of immunogenic cell death-related genes (ICDs) in liver ischemia-reperfusion injury. Through the detailed identification and characterization of these genes, we intend to deepen our insight into the mechanisms underlying LIRI and its relevance to ICD. The discoveries of our research have the potential to open new avenues for therapeutic interventions, which could alleviate the detrimental impacts of LIRI and enhance the prognosis for liver transplant patients.

## 2 Materials and methods

### 2.1 Data download and processing

Expression profiles from the datasets GSE151648, GSE23649, and GSE15480 were downloaded from the Gene Expression Omnibus (GEO, https://www.ncbi.nlm.nih.gov/geo) database. The gene probes were annotated using the R package “hgu133plus2.db” and the genes were conducted log2 transformation. The 34 immunogenic cell death-related genes (ICDs) were retrieved from the published article (18) and listed in **Supplementary Table S1**.

### 2.2 Identification of differentially expressed genes

Differential gene expression analysis was performed using the “limma” R package. The significance threshold was set at *P*-value < 0.05 and logFC > 1 to identify differentially expressed genes (DEGs) between LIRI and control groups. The chromosomal positions of the ICDs were visualized using the “RCircos” R package.

### 2.3 Consensus clustering

Consensus clustering was performed to identify the immunogenic cell death-related molecular subtypes using the “ConcensusClusterPlus” R package. The optimal number of clusters was determined based on the cumulative distribution function (CDF) curve, focusing on relative changes in the area under the curve. To validate the consensus clustering results, the dimensionality reduction technique principal component analysis (PCA) was applied.

### 2.4 GO, KEGG, and GSVA enrichment analysis

Gene Ontology (GO) and Kyoto Encyclopedia of Genes and Genomes (KEGG) enrichment analyses were performed using the R package “clusterProfiler” and “org.Hs.eg.db”. The input genes were first transformed into “ENTREZID” before the enrichment analysis. Moreover, the gene set variation analysis (GSVA) was conducted using the “GSVA” R package by conferring the genes downloaded from the Molecular Signature Database (MSigDB, https://www.gsea-msigdb.org/gsea/msigdb).

### 2.5 Immune infiltration analysis

We performed the CIBERSORT method to analyze the immune cell infiltration of LIRI and normal samples. To better understand the differences in immune infiltration between different LIRI clusters, we employed the single-sample Gene Set Enrichment Analysis (ssGSEA) to assess the immune characteristics. The immune functions were compared between two LIRI clusters.

### 2.6 WGCNA

We conducted a Weighted Gene Co-expression Network Analysis (WGCNA) on the immunogenic cell death-related genes. To identify key modules within the co-expression network, we analyzed the relationships between modules and functional phenotypes. The optimal soft-thresholding power was determined to ensure accurate network construction. Subsequently, we set a height threshold for module detection to identify gene co-expression modules effectively. Additionally, we calculated the person correlation coefficients between each module and the corresponding traits to construct a heatmap of module-trait relationships. The module demonstrating the highest correlation with both gene sets’ enrichment scores was designated as a key module for further investigation. The genes within this key module were subjected to subsequent analyses.

### 2.7 ICD-related model construction based on machine learning algorithms

The four machine learning methods—Random Forest (RF), XGBoost (XGB), Support Vector Machine (SVM), and Generalized Linear Models (GLM) were used to filter the genes and develop the diagnosis model for patients with liver ischemia-reperfusion injury. Receiver operating characteristic (ROC) curves were used to evaluate the performance of the algorithms using the “pROC” R package. The algorithms with the highest area under the curves (AUC) values were selected to construct the models.

### 2.8 Nomogram construction and validation

We constructed the RF-nomogram and SVM-nomogram in the training dataset GSE151648 and verified the accuracy in the validation datasets GSE12720 and GSE23649. ROC curves were utilized to evaluate the predictive ability of the models and genes constituting the RF-nomogram and SVM-nomogram. Calibration curves were created to assess the nomogram’s performance. Additionally, decision curve analysis (DCA) was conducted to analyze the clinical utility of the nomograms.

### 2.9 Liver cell LIRI model

HepG2 cell was purchased from National Collection of Authenticated Cell Cultures (Shanghai, China). Before hypoxia, HepG2 cell was cultured in high-glucose DMEM (11965092, Gibco) with 10% fetal bovine serum (FBS; 1027-106, Gibco) and 1% penicillin and streptomycin (15070063, Gibco) at 37°C in 5% CO_2_. To construct a LIRI model, the medium was replaced with FBS-free and glucose-free DMEM (BL1124A, Biosharp) and cells were transferred to a hypoxia condition (1% O, 5% CO_2_, and 94% N_2_), for 6 h. Then the medium was replaced with high-glucose DMEM and 10% FBS at 37°C in a normoxia condition (5% CO_2_) for 1h.

### 2.10 Real-time quantitative PCR

The total RNA was isolated from HepG2 cell by AG RNAex Pro Reagent (AG21101, Accurate Biology). cDNA was synthesized by the ABScript III RT Master Mix for qPCR with gDNA Remover (RK20429, ABclonal) and real-time quantitative PCR (qPCR) was performed using the 2X Universal SYBR Green Fast qPCR Mix (RK21203, ABclonal). The primers were listed in **Supplementary Table S2**.

### 2.11 Statistical analyses

Statistical analyses were performed using R software (version 4.3.2). Measurement data following a normal distribution was compared using an independent sample t-test. While the non-normally distributed measurement data was analyzed using the Mann–Whitney U test. Correlation analysis was performed using the Spearman method. A *P*-value of <0.05 was considered to be statistically significant.

## 3 Results

### 3.1 Exploring immunogenic cell death-related genes in liver ischemia-reperfusion injury

**Figure 1:**
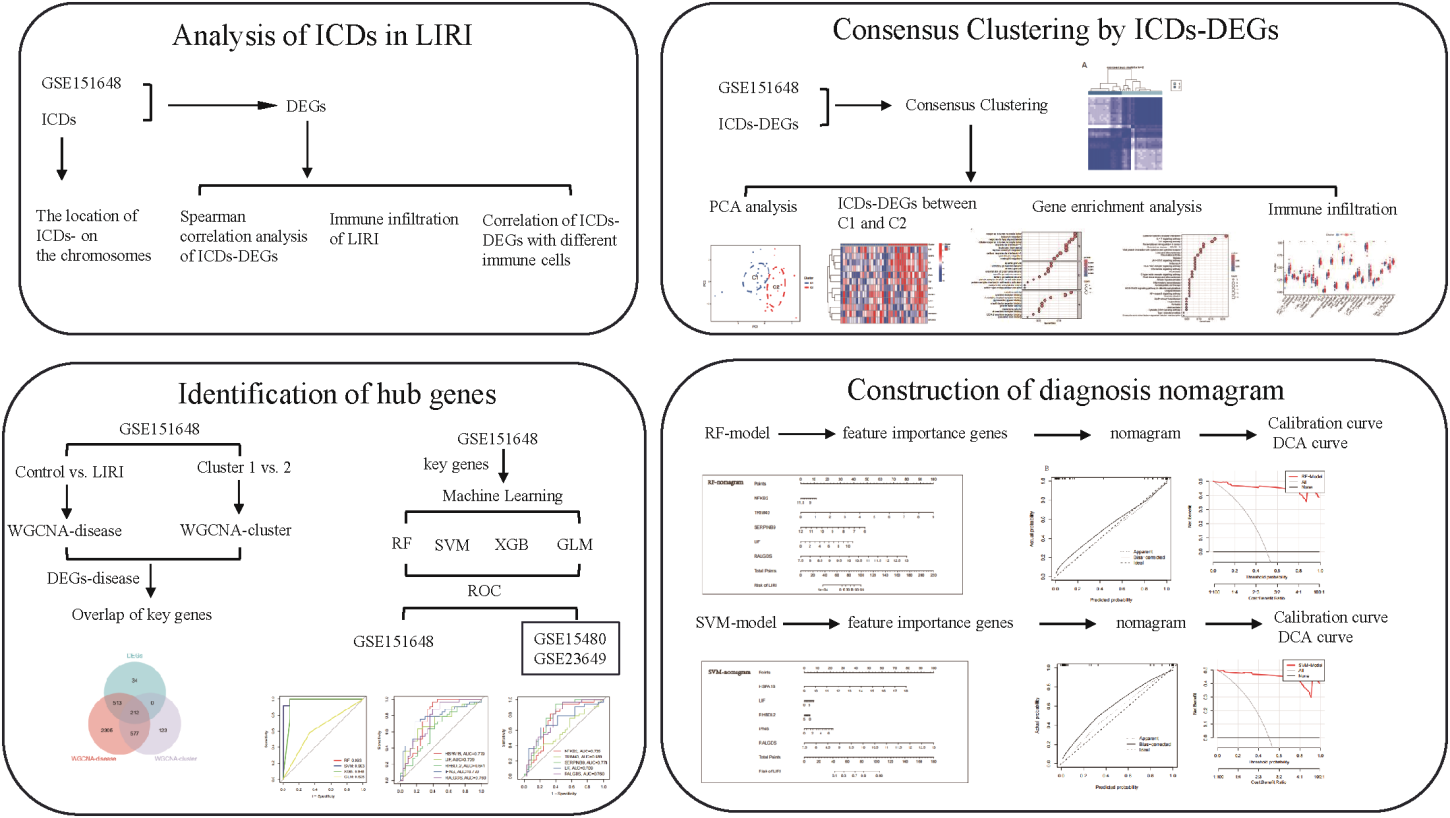
The graphical abstract of our study.

**Figure 2:**
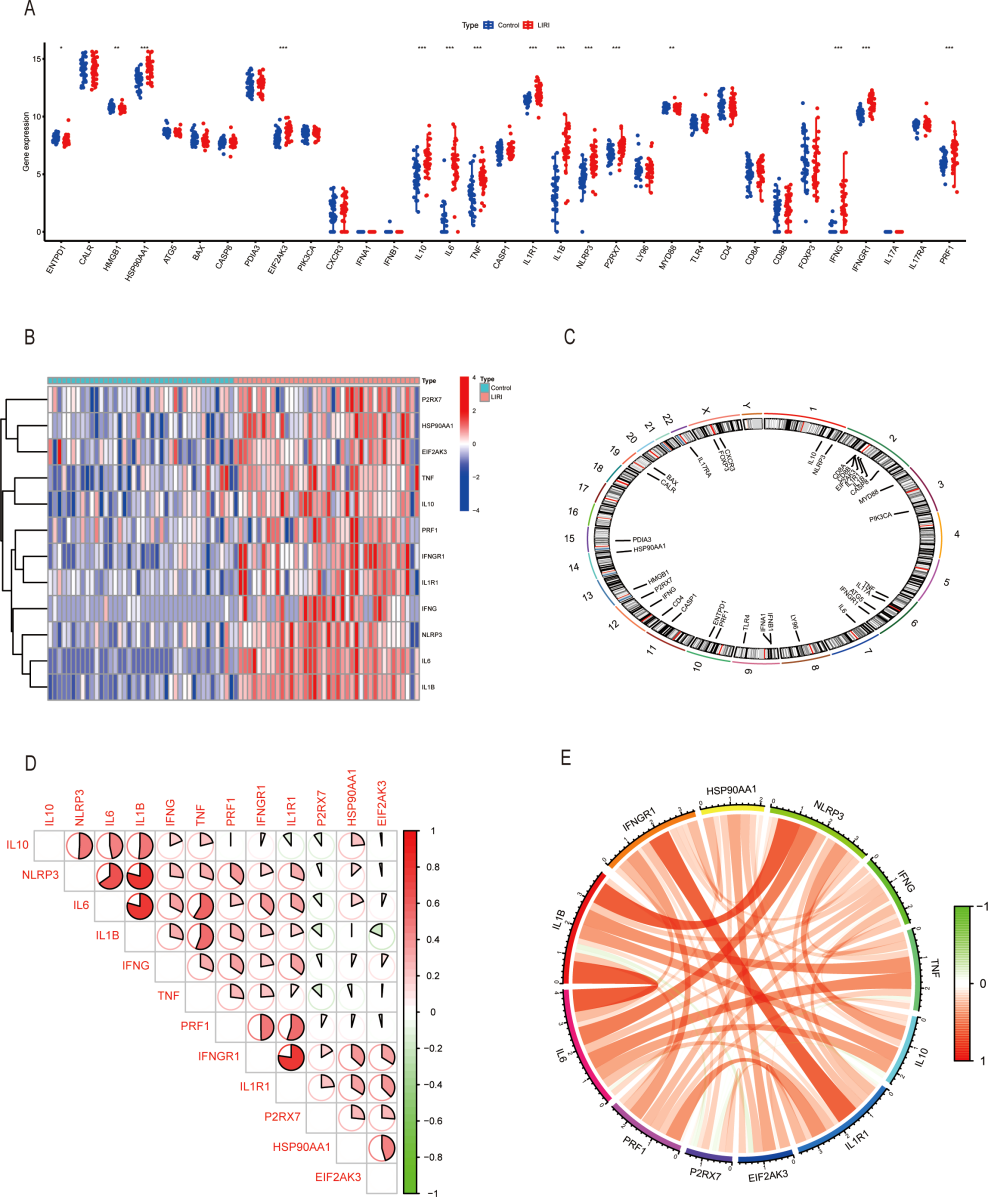
Exploring immunogenic cell death-related genes in liver ischemia-reperfusion injury. **(A)** Boxplot of ICDs in GSE151648. **(B)** Heatmap of ICDs-DEGs in GSE151648. **(C)** The location of ICDs on the chromosomes. **(D, E)** Correlation analysis of ICDs-DEGs by heatmap and circus. *P ≤ 0.05, **P ≤ 0.01, ***P ≤ 0.001.

The overall workflow of our study is summarized in **Figure 1**. We first compared the expression levels of 34 immunogenic cell death-related genes (ICDs) in LIRI samples with normal samples in the GSE151648 dataset. The results showed that there were great differences in the expression levels of ICDs in the two groups (**Figure 2A**). The “limma” R package was used to screen the differentially expressed genes (DEGs) with the threshold set to *P* < 0.05 and |logFC| > 1. The obtained ICDs from the published article are intersected with DEGs of LIRI and normal samples to obtain 12 significant differentially expressed genes related to immunogenic cell death (ICDs-DEGs: P2RX7, HSP90AA1, EIF2AK3, TNF, IL10, PRF1, IFNGR1, IL1R1, IFNG, NLRP3, IL6, and IL1B). The “pheatmap” R package was used to plot a heatmap of the expression levels of ICDs-DEGs (**Figure 2B**). Moreover, we also visualized the locations of ICDs in the chromosome (**Figure 2C**). The correlation pie chart (**Figure 2D**) and the chord diagram (**Figure 2E**) further showed the tight correlations between ICDs-DEGs.

### 3.2 Immune cell infiltration analyses of LIRI by CIBERSORT

**Figure 3:**
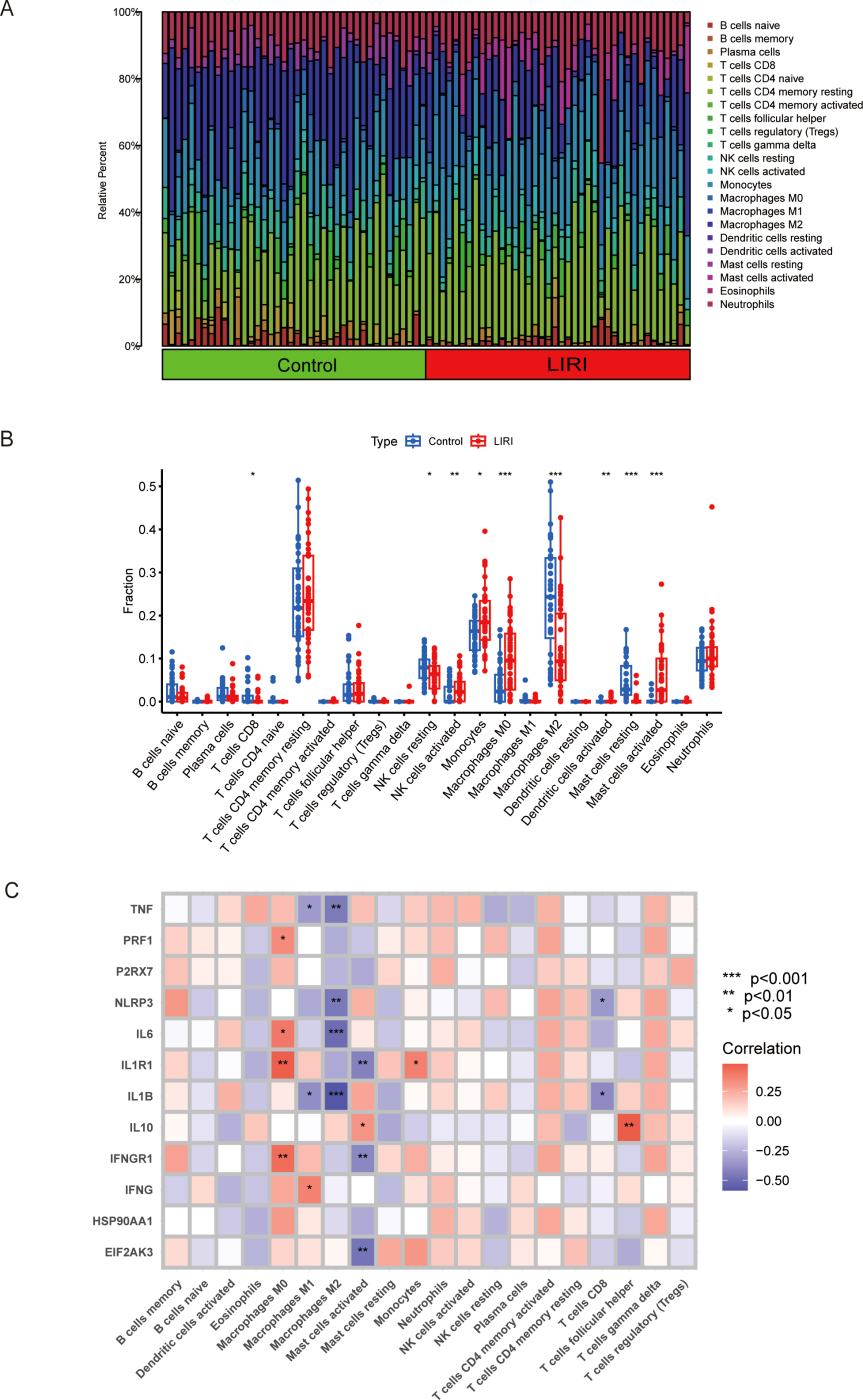
Analysis of immune infiltration in liver ischemia-reperfusion injury by CIBERSOFT. **(A, B)** Boxplot of immune infiltration between control and LIRI. **(C)** Correlation of ICDs-DEGs with different immune cells by heatmap. *P ≤ 0.05, **P ≤ 0.01, ***P ≤ 0.001.

Growing evidence has proven that the tumor microenvironment (TME) is closely related to the disease progression and therapy response (20). To better understand the immune features of the liver ischemia-reperfusion injury, we conducted the immune cell infiltration analysis using the CIBERSORT method. The results showed that the proportions of the immune cells were higher in LIRI samples than in normal samples (**Figure 3A**). We performed comparisons of immune cells in two groups and found that there were higher NK cells activated, monocytes, macrophage M0, dendritic cells activated, and mast cells activated; lower T cells CD8, NK cells resting, macrophages M2, and mast cells resting in LIRI group than in normal group (**Figure 3B**). Our study also analyzed the relationship between the expression of pivotal ICDs-DEGs and immune cell components. The result showed that the expression levels of ICDs-DEGs had significant connections with immune proportions (**Figure 3C**). As a result, investigating the mechanisms of immunogenic cell death could help understand the immune functions of LIRI thus providing new clues for the prevention and treatment of LIRI complications.

### 3.3 Consensus clustering based on the expression of ICDs-DEGs

**Figure 4:**
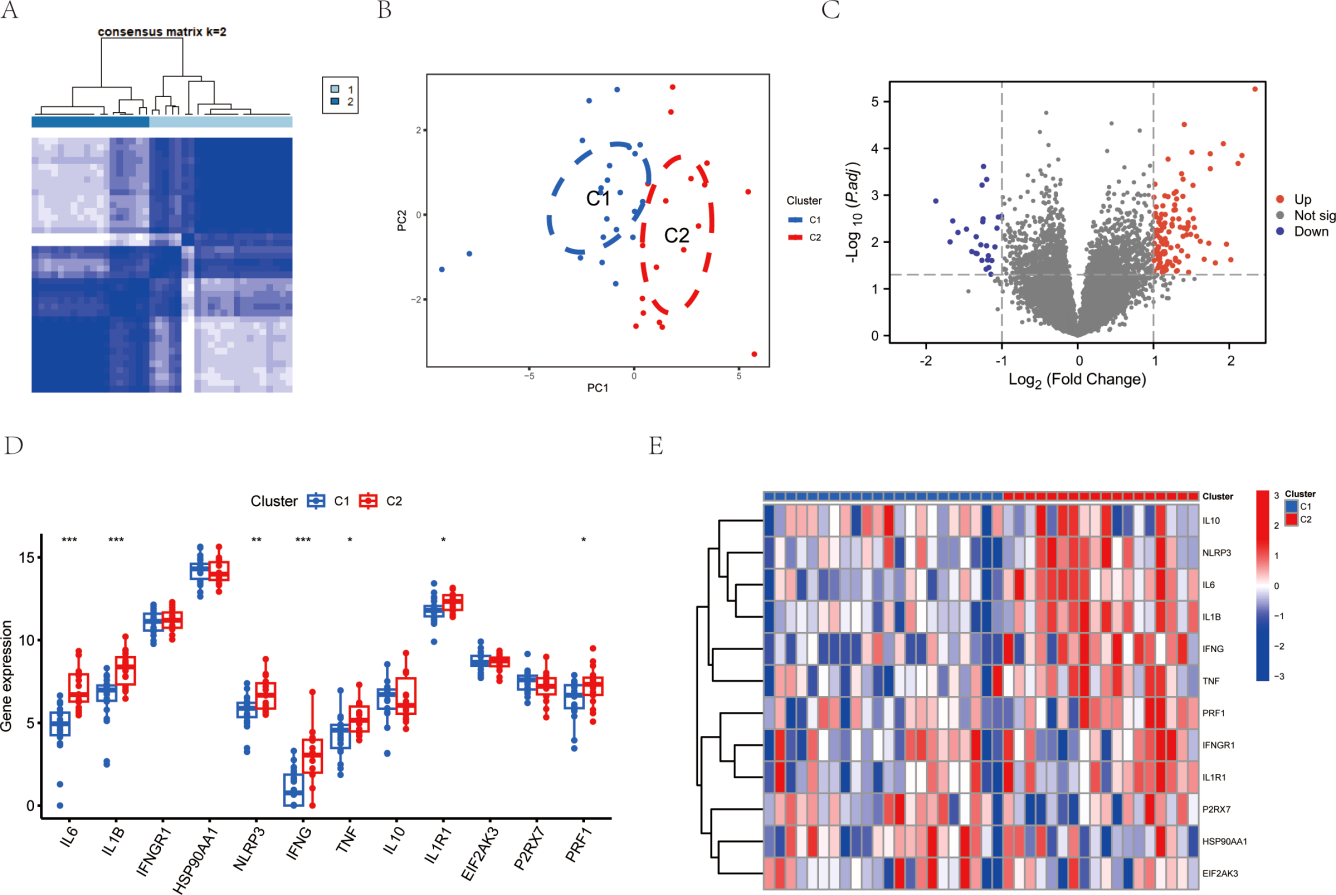
Consensus clustering analysis of LIRI by ICDs-DEGs. **(A)** Consensus clustering matrixes were generated for values of k=2. **(B)** PCA analysis of Cluster 1 and Cluster 2. **(C)** Volcano plot of gene expression levels between Cluster 1 and Cluster 2. **(D)** Boxplot of ICDs-DEGs between Cluster 1 and Cluster 2. **(E)** Heatmap of ICDs-DEGs between Cluster 1 and Cluster 2. *P ≤ 0.05, **P ≤ 0.01, ***P ≤ 0.001.

To analyze the mechanisms of immunogenic cell death in ischemic reperfusion injury, we conducted consensus clustering based on the expression levels of ICDs-DEGs. The results showed that the number of clusters k=2 achieved the best clustering effect (**Figure 4A**). Then the samples of GSE151648 were divided into Cluster 1 and Cluster 2 based on the clustering algorithm. The main component analysis showed that the two clusters could be separated from each other (**Figure 4B**). Subsequently, we identified the differentially expressed genes of the two clusters and visualized them using the volcano plot (**Figure 4C**). In terms of the hub gene expression signatures, there were significant differences in the ICDs-DEGs between Cluster 1 and Cluster 2. To be more specific, Cluster 1 has higher gene expression levels of IL6, IL1B, NLRP3, IFNG, TNF, IL1R1, and PRF1 (**Figure 4D**). The heatmap graphically showed that the ICDs-DEGs expressed more in Cluster 2 than in Cluster 1 (**Figure 4E**).

### 3.4 Pathway enrichment analysis of the two clusters

**Figure 5:**
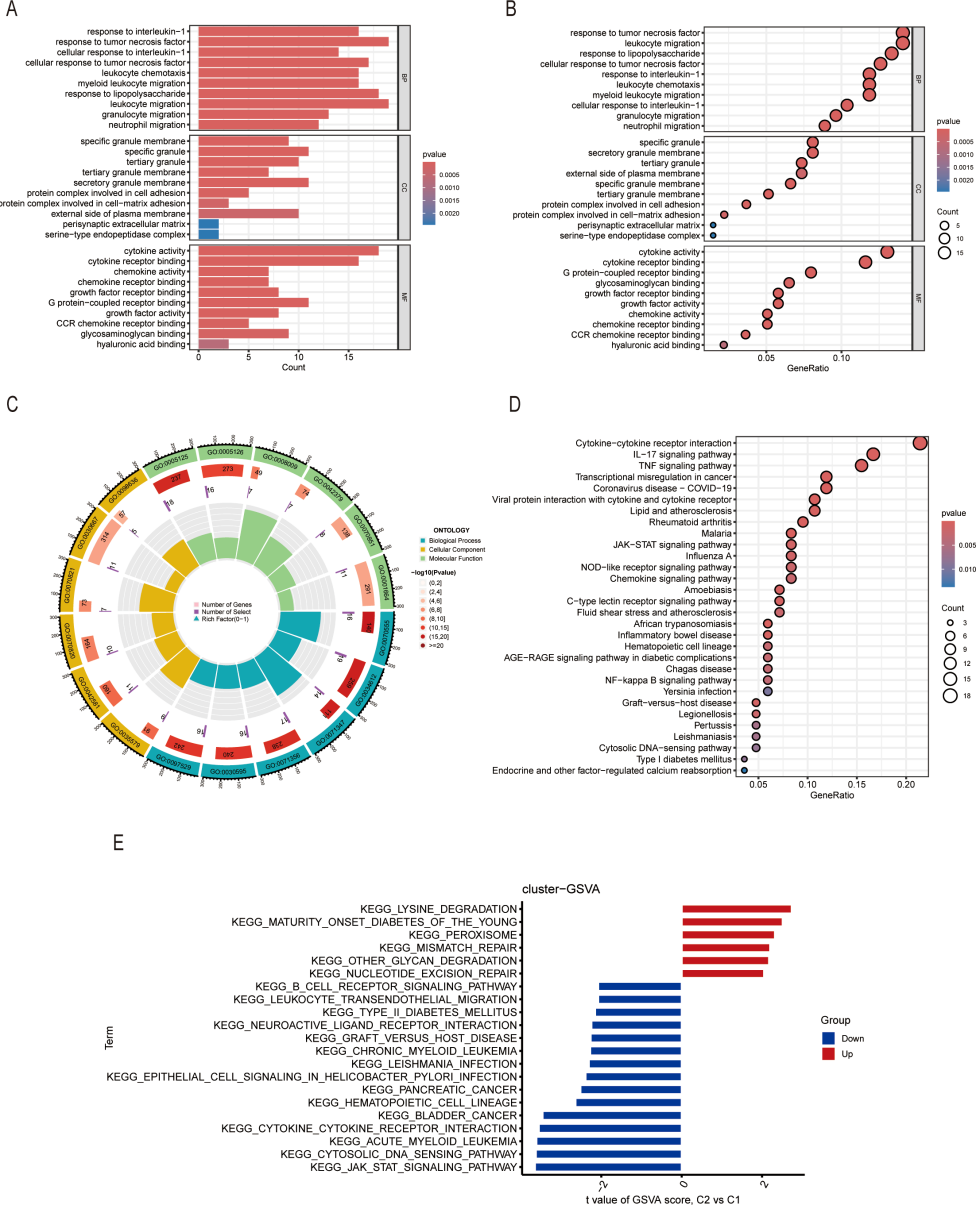
Gene enrichment analysis of DEGs between two clusters. **(A-C)** The column plots, bubble plots, and circus represent the GO enrichment of DEGs, including BP, CC, and MF. **(D)** KEGG enrichment of DEGs. **(E)** GSVA enrichment of DEGs.

We also analyzed the DEGs between Cluster 1 and Cluster 2 and performed the pathway enrichment analyses of the two clusters. GO enrichment analysis showed that there were several pathways enriched. The bubble and bar charts as well as the chord diagram all showed that DEGs of the two clusters were mainly enriched in response to interleukin-1, tumor necrosis factor, and leukocyte migration signaling pathway in the biological process (BP); in the cellular component (CC), DEGs were mainly enriched in granule-related pathways; in the molecular function (MF), DEGs were enriched primarily in cytokine activity and cytokine receptor binding pathways (**Figures 5A-C**). On the other hand, the KEGG pathway enrichment analysis showed that there were similar pathways enriched including the cytokine-cytokine receptor interaction, IL-17 signaling pathway and TNF signaling pathway (**Figure 5D**). The GSVA result also revealed that the DEGs were related to several immune-associated pathways shown specifically in **Figure 5E**. In conclusion, these results all shed light on the important role of ICDs in the molecular clustering of liver ischemia-reperfusion injury.

### 3.5 Immune infiltration analyses of the two clusters by ssGSEA

**Figure 6:**
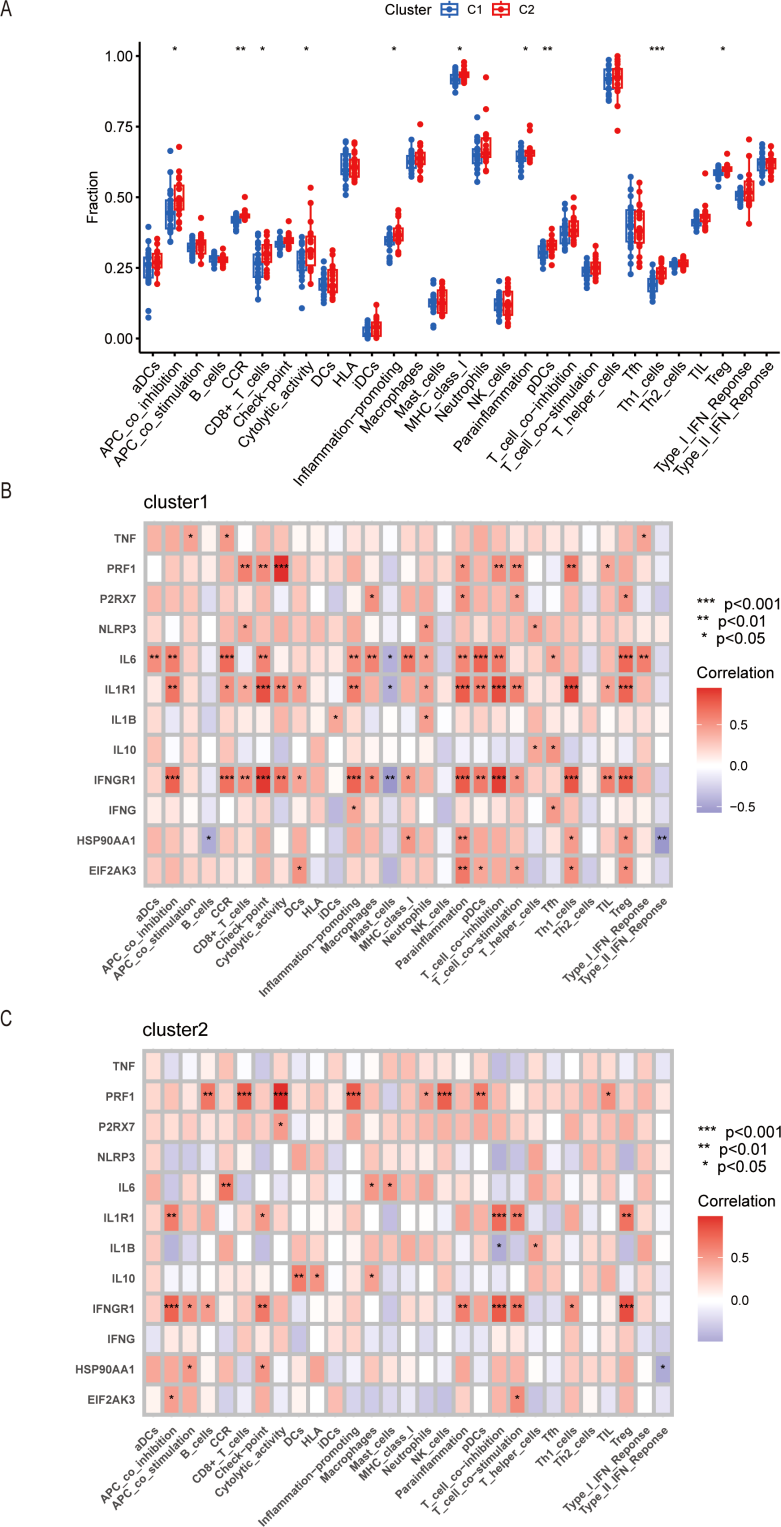
Immune infiltration in Cluster 1 and Cluster 2 by ssGSEA. **(A)** Boxplot of different immune cells or processes between Cluster 1 and Cluster 2. **(B)** Correlation of ICDs-DEGs with immune infiltration in Cluster 1. **(C)** Correlation of ICDs-DEGs with immune infiltration in Cluster 2. *P ≤ 0.05, **P ≤ 0.01, ***P ≤ 0.001.

To investigate the immune functions of the two clusters, we conducted the ssGSEA analysis. The results showed that the infiltration of immune functions was different between Cluster 1 and Cluster 2. We performed the comparisons between immune cells and found that there were higher in APC_co-inhibition, CCR, CD8+_T_cells, cytolytic activity, inflammation-promoting, MHC_class_I, para-inflammation, pDCs, Th1_cells, and Treg in Cluster 2 (**Figure 6A**), which proved that the Cluster 2 had a high level of immune state. Our study also analyzed the relationship between the expression of ICDs-DEGs and immune components in the two clusters. The results showed that the expression level of PRF1 had more connections with immune proportions in Cluster 2; while the expression levels of IL6, IL1R1, and IFNGR1 had tighter relationships with immune cells in Cluster 1 (**Figures 6B, C**).

### 3.6 Identification of the hub genes in liver ischemia-reperfusion injury by WGCNA

**Figure 7:**
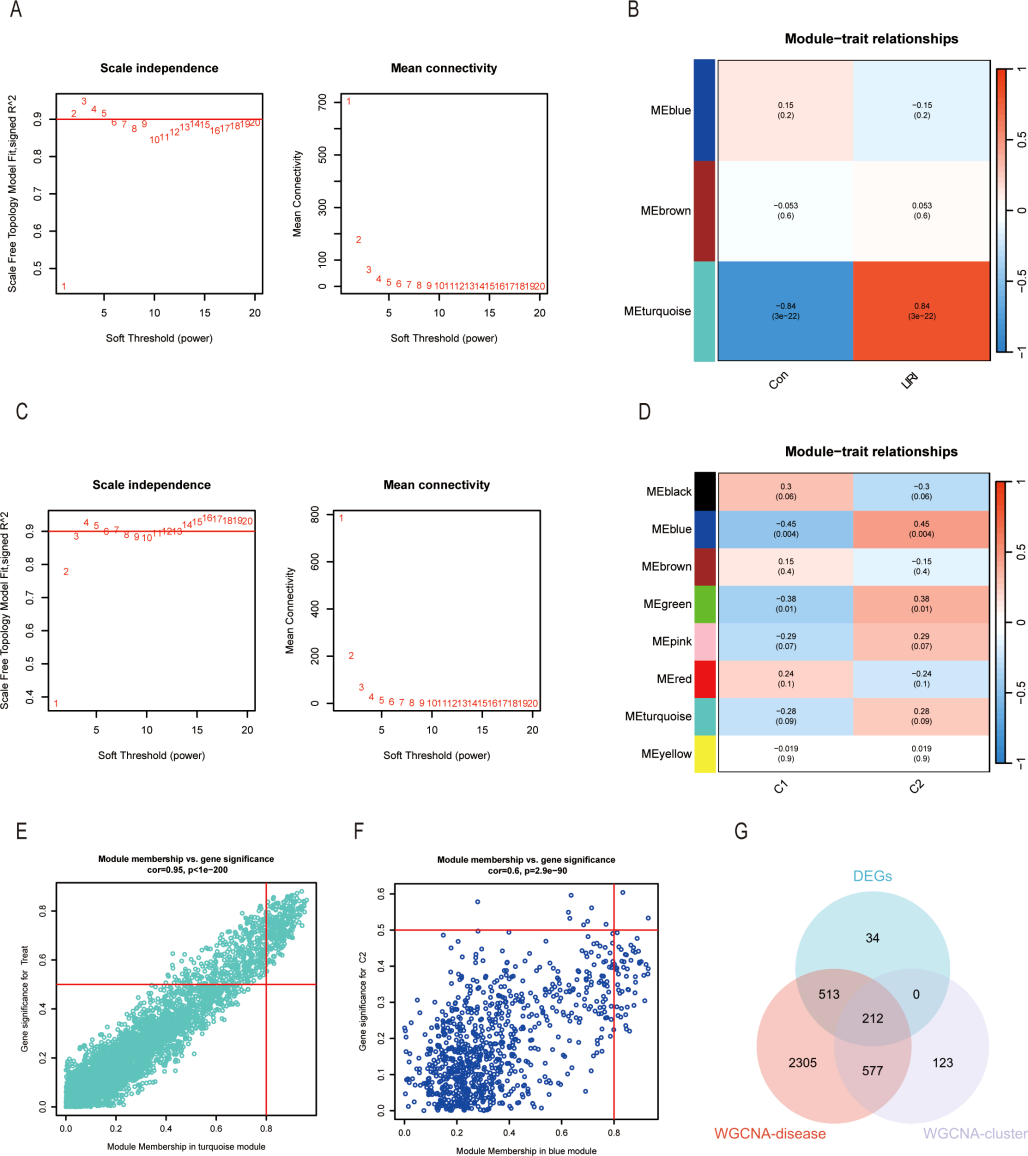
Identification of gene modules associated with LIRI and ICD by WGCNA. **(A)** The selection of optimal soft thresholding power (β) between control and LIRI. The scale-free fit index (left) and mean connectivity (right) for different soft-thresholding powers. **(B)** The correlation heatmap between different gene modules and status of LIRI. **(C)** The selection of optimal soft thresholding power (β) between different clusters. The scale-free fit index (left) and mean connectivity (right) for different soft-thresholding powers. **(D)** The correlation heatmap between different gene modules and the status of different clusters. **(E)** Scatter plots showing the correlation between module membership and gene significance in the turquoise module of LIRI and blue module of different clusters. **(F)** Venn diagram of key modules and DEGs from LIRI.

The WGCNA method was used to identify the hub genes in hepatic ischemic reperfusion injury. We first analyzed the co-expression network in LIRI samples and normal samples. A gene hierarchical clustering dendrogram was constructed based on gene correlations. The soft threshold was set at 3 to achieve a scale-free topology for the network, yielding an R² value of 0.9 and high average connectivity (**Figure 7A**). Ultimately, we determined that the “turquoise” module, comprising 3,607 genes, was the significant module in LIRI (**Figure 7B**). The scatter plot showed a strong correlation between the module membership in turquoise and the gene significance for LIRI (**Figure 7E**, Cor = 0.95, *P* < 1e−200). Likewise, we performed the WGCNA in Cluster 1 and Cluster 2 to filter the most relevant genes of ICD. The blue module containing 912 genes was selected based on the analyses (**Figures 7C, D**). The correlation coefficient was 0.6 for the module membership in blue and the gene significance for Cluster 2 (**Figure 7F**, *P* = 2.9e−90). In the end, we took the intersection of the DEGs between LIRI and normal samples, the WGCNA-disease genes of the LIRI, and the WGCNA-cluster genes to identify the hub genes highly associated with ICD and LIRI (**Figure 7G**).

### 3.7 Construction of the diagnosis model by machine learning

**Figure 8:**
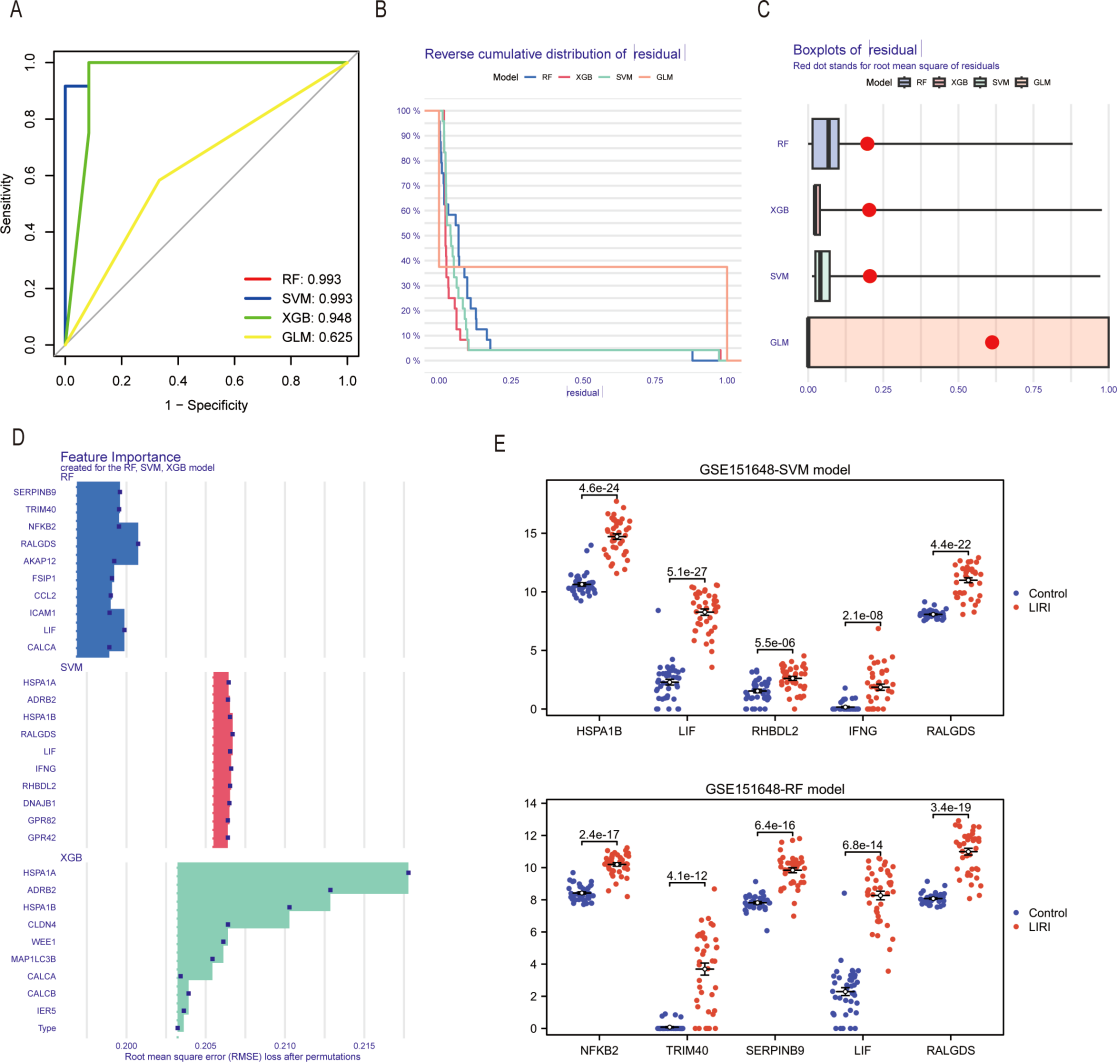
Identification of hub genes in LIRI by machine learning. **(A)** The ROC curves of different machine learning models in the GSE151648. **(B)** The reverse cumulative distribution curves of residual in different machine learning methods. **(C)** Boxplot of residuals in different machine learning models. **(D)** Feature importance genes created for the RF and SVM model. **(E)** Differential expression analysis of feature importance genes from RF and SVM models in GSE151648.

Four machine learning algorithms were utilized to analyze the dimensionality reduction of the 212 genes screened above. The ROC results showed the area under the curves (AUC) of the four machine learning algorithms. (**Figure 8A**). The reverse cumulative distribution and boxplots of residual were plotted to show the residual distribution of the models (**Figures 8B, C**). Because of the high residual, we dismissed the GLM model. Moreover, It proved that the root mean square error (RMSE) loss after permutations was smaller in the RF and SVM models than in the XGB model (**Figure 8D**). Since the RF and SVM models had the same AUC of 0.993, we selected both for the construction of the diagnosis model of liver ischemia-reperfusion injury. The results showed that the ICDs constructing the model had higher expression levels in the LIRI group than in the normal group (**Figure 8E**).

**Figure 9:**
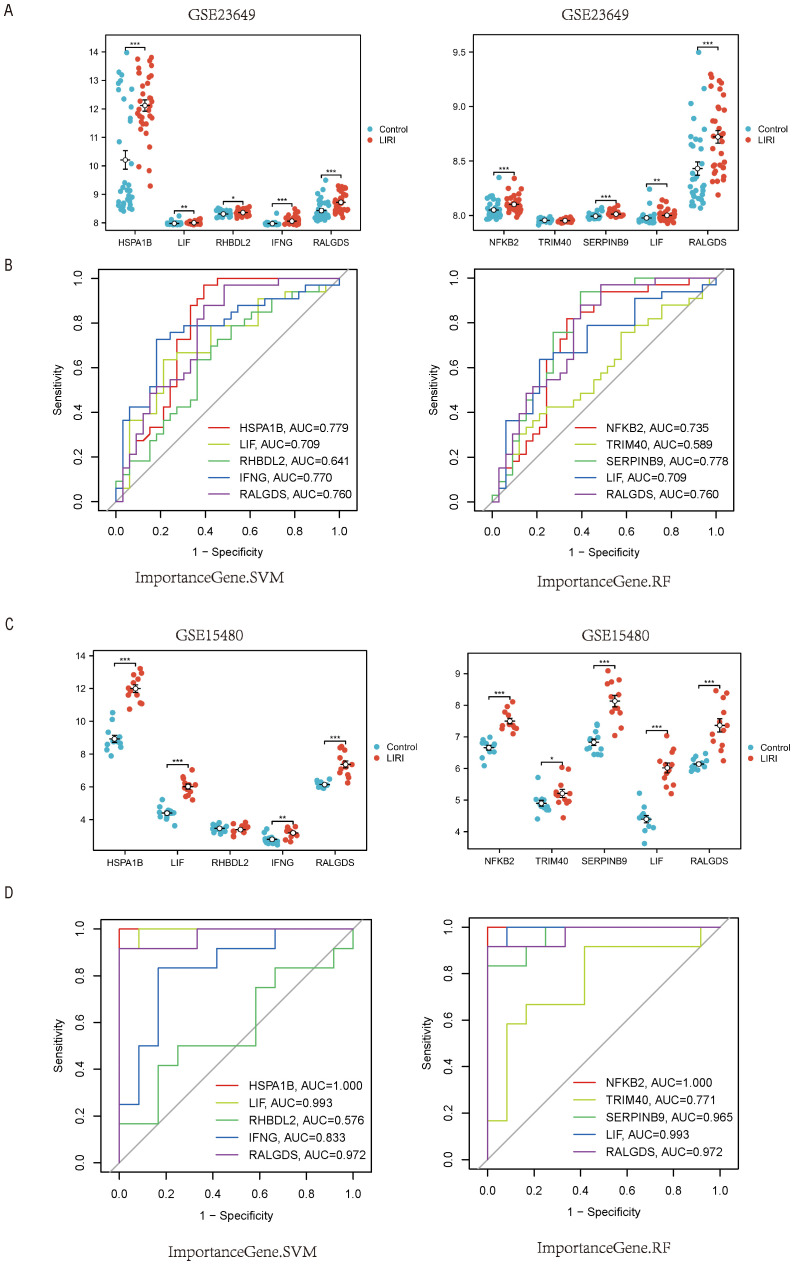
External validation of the hub genes in LIRI. **(A)** Boxplot of the expression of feature importance genes from RF and SVM in GSE23649. **(B)** The ROC curves of feature importance genes from RF and SVM in GSE23649. **(C)** Boxplot of the expression of feature importance genes from RF and SVM in GSE15480. **(D)** The ROC curves of feature Importance genes from RF and SVM in GSE15480.

To further verify the models, we chose the GSE23649 and GSE15480 as the validation sets. Our results proved that the genes constituting the RF and SVM models also had higher expression levels in the LIRI group than in the normal group of the validation cohort GSE23649. In addition, the AUCs of the model genes had excellent performances in GSE23649 (**Figures 9A, B**). The same conclusion also arrived in the validation cohort GSE15480 (**Figures 9C, D**).

### 3.8 Construction of the nomograms

**Figure 10:**
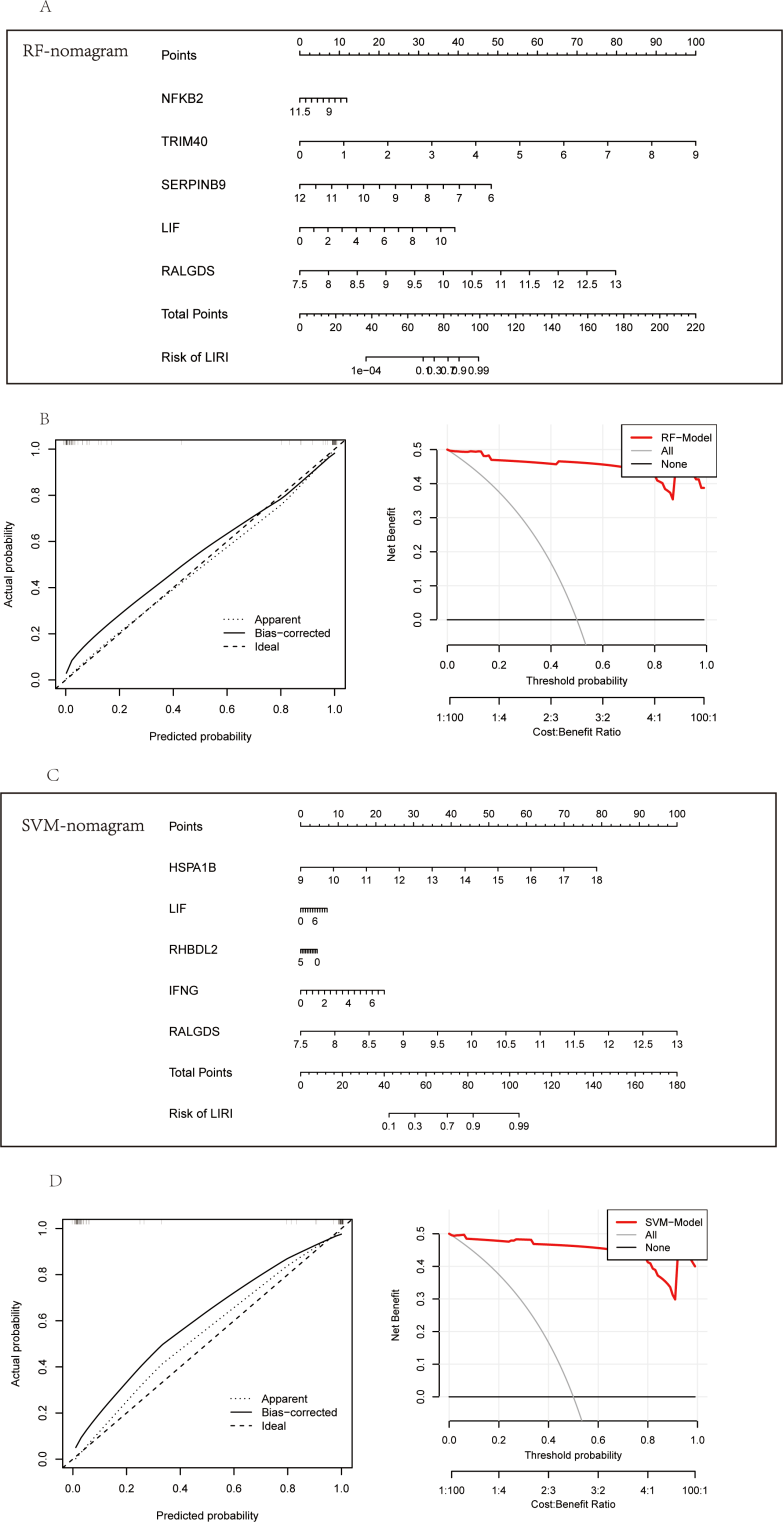
Construction of the diagnostic nomogram in LIRI. **(A)** Nomogram of feature importance genes from RF. **(B)** The calibration and DCA curves of the nomogram based on RF feature importance genes. **(C)** Nomogram of feature Importance genes from SVM. **(D)** The calibration and DCA curves of the nomogram based on SVM feature importance genes.

Considering the superior performance of the diagnosis models, we constructed the nomograms to further validate the clinical applicability of the models. The nomograms showed the relationship of the hub genes constituting the model and the risk of LIRI (**Figures 10A, C**). Calibration curves of the nomograms demonstrated remarkable alignments between the predicted values and actual probabilities, providing support for the reliability of the diagnosis models (**Figures 10B, D**; left). In addition, the decision curve analysis (DCA) curves proved the high clinical values of the nomograms to diagnose LIRI in patients (**Figures 10B, D**; right).

### 3.9 The expression of key genes in a cell LIRI model

**Figure 11:**
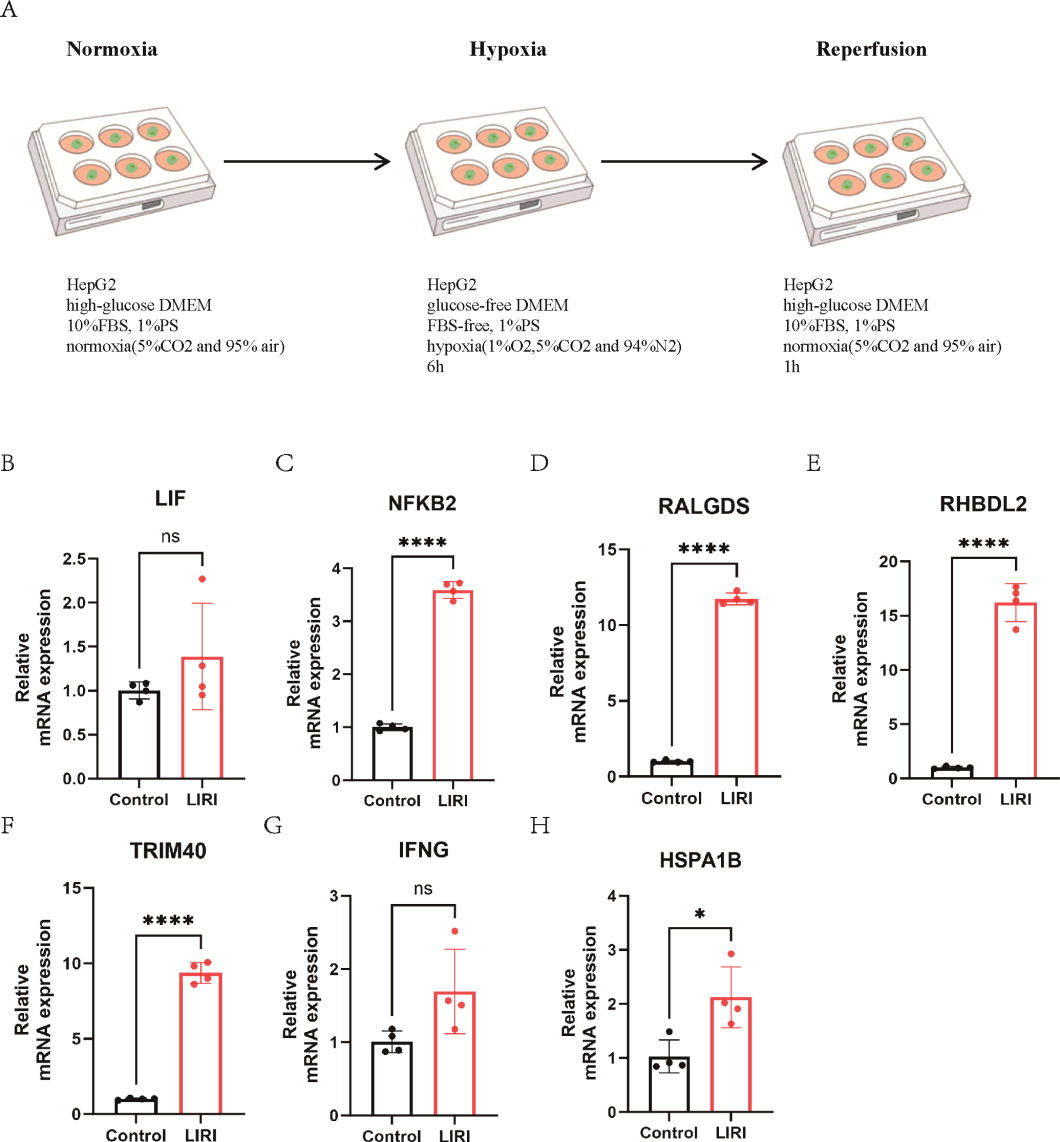
The expression of key genes in a cell LIRI model. **(A)** The graphical abstract of LIRI model. **(B-H)** The mRNA expression of key genes from RF and SVM model. ns P > 0.05, * P ≤ 0.05, **** P ≤ 0.0001.

We have constructed RF-model and SVM-model for the diagnosis of LIRI, which showed relatively high diagnostic value. Hence, we constructed a cell LIRI model to further verify the key genes in RF-model and SVM-model. In LIRI model, HepG2 cell was cultured in a hypoxia condition for 6h and then transferred to a normoxia condition for 1h (**Figure 11A**). Then mRNA expression of key genes from RF-model and SVM-model were detected by qPCR. Consistent with the results of bioinformatics analysis, the mRNA expression of NFKB2, RALGDS, RHBDL2, TRIM40 and HSPA1B were upregulated in LIRI model (**Figures 11C–F, H**). However, the expression of LIF and IFNG showed no significant difference between Control and LIRI (**Figures 11B, G**).

## 4 Discussion

The liver ischemia-reperfusion injury (LIRI) poses a significant challenge in the context of hepatic resection and transplantation (21). LIRI of the liver is a major cause of hepatic dysfunction following liver transplantation, highlighting the need for effective strategies to prevent this liver damage (22). Understanding the mechanisms of LIRI is essential for developing therapeutic interventions.

LIRI has two distinct stages including ischemia and reperfusion (23). The ischemic phase is characterized by metabolic disorders in local tissue cells, including the continuous depletion of glycogen, oxygen deficiency, and adenosine triphosphate (ATP) depletion, leading to the death of liver parenchymal cells. Reperfusion follows the ischemic phase and is not only marked by metabolic disorders but also by immune responses (24). Therefore, a thorough investigation of the immunogenic cell death occurring in LIRI is essential for diagnosing and uncovering new therapeutic strategies for LIRI, such as inhibiting harmful pro-inflammatory reactions or promoting beneficial anti-inflammatory responses.

In this research, we analyzed the core mechanisms of ICDs in liver ischemia-reperfusion injury and constructed machine learning-based diagnosis models for LIRI. We analyzed the expression of ICDs in LIRI samples and normal samples. We found that the levels of ICDs were higher in LIRI, which hints that ICD could play an important role in liver ischemia-reperfusion injury. Cell death can be immunogenic based on four crucial factors. First, cytotoxicity requires cells to undergo stress responses before dying. Second, antigenicity involves the expression of recognizable antigens by T cells. Third, adjuvanticity entails the release of danger-associated molecular patterns (DAMPs) that facilitate dendritic cell (DC) recruitment and maturation, enabling effective antigen presentation to T lymphocytes. Fourth, a permissive microenvironment must allow access to both DCs and T cells (17, 19, 25).

In conclusion, the published research indicated that LIRI had a tight connection to the immune process. Therefore, we analyzed the immune cell infiltration of LIRI and normal liver samples and found that the proportion of immune cells was different in LIRI and normal samples. To be specific, the infiltration of NK cells activated, monocytes, macrophage M0, dendritic cells activated, and mast cells activated were all higher in LIRI, which suggested an intensified innate immune reaction to tissue damage. In our research, we also performed consensus clustering to identify two subtypes of LIRI. We also analyzed the immune functions of Cluster 1 and Cluster 2 using the ssGSEA method. We found that the proportions of immune functions were higher in Cluster 2 than in Cluster 1, indicating that Cluster 2 had a more activated inflammatory microenvironment. Moreover, the pathway enrichment results proved that the DEGs of the two clusters were highly enriched in cytokine and chemokine-related pathways, suggesting the important role of these molecules in immunogenic cell death.

It has been reported that the liver ischemia-reperfusion injury (LIRI) can be classified into two types: warm LIRI, resulting from hepatocyte damage during *in vivo* liver transplantation, potentially leading to liver failure; cold LIRI, caused by damage to hepatic cells during *ex vivo* preservation, which is often followed by warm LIRI during transplantation (26). Despite different initial cell death mechanisms, both types share similar pathophysiological processes mediated by innate immune responses, which involve the activation of macrophages and neutrophils (27, 28), production of cytokines and chemokines (29), release of reactive oxygen species (ROS) (30), and infiltration of lymphocytes or monocytes (31). Our immune infiltration and pathway enrichment analyses aligned with the existing literature, highlighting the pivotal role of ICD in ischemia-reperfusion injury, which could yield novel perspectives on treatment after liver transplantation.

In the end, we constructed the ICD-related diagnosis models based on the WGCNA method and four machine learning algorithms. The model had excellent performances in both the training and validation cohorts. The nomograms proved that our diagnosis models could help predict the risk of LIRI after liver transplantation.

To validate the essential genes identified through machine learning, we established an *in vitro* model of ischemia-reperfusion in HepG2 cell and performed qPCR experiments. The findings indicated that the mRNA expression of NFKB2, RALGDS, RHBDL2, TRIM40 and HSPA1B were increased. The NF-κB signaling pathway plays a critical role in the regulation of oxidative stress, inflammatory responses, apoptosis, and mitochondrial dysfunction, and is intricately linked to the pathophysiological mechanisms underlying LIRI (32–34). A growing body of research highlights the potential therapeutic efficacy of pharmacological agents or inhibitors that specifically target NF-κB in the management of LIRI (35–37). RALGDS functions as a guanine nucleotide exchange factor for the small G protein Ral and is classified as one of the Ras effectors. It plays a critical role in the regulation of membrane transport and the remodeling of the cytoskeleton (38, 39). RHBDL2 is a member of the rhomboid family of the integral membrane proteins and functions as an intramembrane serine protease (40, 41). TRIM40 is a member of the tripartite motif-containing protein (TRIM) protein family. A previous study showed that the upregulated TRIM40 could promote the progression of inflammatory bowel disease. TRIM40 is a pathogenic driver of inflammatory bowel disease through subverting intestinal barrier integrity (42). However, as of now, there is a lack of research publications concerning RALGDS, RHBDL2 and TRIM40 within the context of LIRI. HSPA1B is a member of the heat shock protein (HSP) family. As a protein induced under various environmental stresses such as high temperature, hypoxia, chemicals, and oxidative stress, its main function is to protect cells from damage caused by these stresses (43, 44). Previous studies have reported that HSPA12A in hepatocytes inhibits macrophage chemotaxis and activation by suppressing glycolysis-mediated HMGB1 lactylation and hepatocyte secretion, thereby alleviating liver ischemia/reperfusion injury (27). In this article, through bioinformatics and the LIRI cell model, we found that HSPA1B was significantly elevated; however, the precise mechanisms underlying its role in LIRI require further investigation.

Nonetheless, there are also several limitations in our research. First, our research mainly relies on bioinformatic analysis, and the conclusion has not been validated by biological experiments. Second, due to the limited diversity of non-oncology databases, the sequencing data of LIRI is constrained solely to the GEO database. Our future studies will focus on delineating specific molecular pathways, validating the hub genes involved in the model, and incorporating data from additional sources that could enhance reliability.

## 5 Conclusions

In conclusion, our comprehensive analyses underscored the intricate relationship between immunogenic cell death and ischemia-reperfusion injury after liver transplantation. Moreover, we also identified two clusters by consensus clustering which had different immune infiltration degrees. Finally, we constructed the ICD-related diagnosis models based on WGCNA and machine learning algorithms. Our RF and SVM-based models had excellent performances in the diagnosis of ischemia-reperfusion injury.

## Data Availability

The original contributions presented in the study are included in the article/**Supplementary Material**. Further inquiries can be directed to the corresponding authors.

## Glossary

ATP: adenosine triphosphate
AUC: area under the curves
BP: biological process
CC: cellular component
CDF: cumulative distribution function
DAMPs: damage-associated molecular patterns
DC: dendritic cell
DCA: decision curve analysis
DEGs: differentially expressed genes
GEO: Gene Expression Omnibus
GLM: Generalized Linear Models
GSVA: gene set variation analysis
HSPA1B: Heat Shock Protein Family A (Hsp70) Member 1B
ICD: immunogenic cell death
ICDs: immunogenic cell death-related genes
ICDs-DEGs: differentially expressed genes related to immunogenic cell death
IFNG: Interferon-gamma
KEGG: Kyoto Encyclopedia of Genes and Genomes
LIF: Leukemia inhibitory factor
LIRI: liver ischemia-reperfusion injury
MF: molecular function
NFKB2: Nuclear Factor Kappa B Subunit 2
PCA: principal component analysis
RF: Random Forest
RALGDS: Ral Guanine Nucleotide Dissociation Stimulator
RHBDL2: Rhomboid Like 2
RMSE: root mean square error;ROC, receiver operating characteristic
ROS: reactive oxygen species
ssGSEA: single sample gene set enrichment analysis
SVM: Support Vector Machine
TME: tumor microenvironment
TRIM40: Tripartite Motif Containing 40
WGCNA: Weighted Gene Co-expression Network Analysis
XGB: XGBoost
